# Rapid Multiplex Genotyping of 20 HLA-A^*^02:01 Restricted Minor Histocompatibility Antigens

**DOI:** 10.3389/fimmu.2019.01226

**Published:** 2019-06-04

**Authors:** Dmitrii S. Romaniuk, Anna M. Postovskaya, Alexandra A. Khmelevskaya, Dmitry B. Malko, Grigory A. Efimov

**Affiliations:** Laboratory for Transplantation Immunology, National Research Center for Hematology, Moscow, Russia

**Keywords:** multiplex qPCR, minor histocompatibility antigens, MiHA, SNP genotyping, allele-specific primer, AS-PCR, HSCT, AS-qPCR

## Abstract

A subset of MHC-associated self-peptides presented by the recipient's cells and immunologically foreign to the donor can induce an allogeneic immune response after hematopoietic stem cell transplantation (HSCT). These immunogenic peptides originate from the genomic polymorphisms and are known as minor histocompatibility antigens (MiHA). MiHA mismatches trigger the post-transplant immune response, which could manifest in both the deleterious “graft-vs.-host” disease and the beneficial “graft-vs.-leukemia” effect. Importantly, some MiHAs are considered to be promising targets for posttransplant T-cell immunotherapy of hematopoietic malignancies. This creates a demand for a robust and fast approach to genotyping MiHA-encoding polymorphisms. We report a multiplex real-time PCR method for the genotyping of 20 polymorphisms that are encoding HLA-A^*^02:01-restricted MiHAs. This method uses allele-specific primers and gene-specific hydrolysis probes. In 1 h it allows for the detection of MiHA mismatches in a donor-recipient pair without the need for electrophoresis, sequencing, or other time-consuming techniques. We validated the method with Sanger and NGS sequencing and demonstrated good performance over a wide range of DNA concentrations. We propose our protocol as a fast and accurate method of identifying mismatched MiHAs. The information on the MiHA mismatches is useful for studying the allogeneic immune response following HSCT and for selecting the targets for post-transplant T-cell therapy.

## Introduction

Allogeneic hematopoietic stem cell transplantation (allo-HSCT) is commonly used as a treatment for acute leukemias, lymphomas and other malignant hematopoietic diseases. The therapeutic efficiency of HSCT is determined by immune recognition and subsequent elimination of the remaining malignant clone by the infused lymphocytes of the donor origin, or so-called “graft-vs.-leukemia” (GvL) effect (1). Unfortunately, donor lymphocytes can also recognize and target some healthy non-hematopoietic tissue antigens, triggering potentially lethal “graft-vs.-host” disease (GvHD) (2).

The targets of the alloreactive immune response in the HLA-matched allo-HSCT are the minor histocompatibility antigens (MiHA). MiHAs are endogenous polymorphic peptides, presented by MHC molecules on the cell surface (3, 4). Donor T cells were not selected to tolerate the recipient's MiHA alleles, thus some transplanted T-cell clones may recognize them as foreign antigens (5, 6). Most MiHAs originate from non-synonymous single-nucleotide polymorphisms (nsSNP), however, they can be derived from other polymorphism types. Frameshifts and nonsense mutations result in the expression of truncated proteins, so only one of the allelic variants encodes a peptide, presented by MHC (7). Even a single amino acid substitution, caused by missense SNP, can affect peptide cleavage by the proteasome (8) or the antigen processing, resulting in the unilateral presentation of one of the allelic variants (9). The SNP variants encoding the presented and non-presented peptides are denoted as the immunogenic (dominant) and non-immunogenic (recessive) alleles, respectively. For the immune reaction to develop, the recipient should have at least one dominant allele, while the donor needs to have two copies of the recessive allele. For some MiHAs both alleles encode MHC-presented peptides, potentially making them immunogenic in both directions (co-dominant). In this case, T-cells discriminate peptides by a single amino acid difference (10). Up to date over 60 MiHAs have been discovered. For the majority of their respective allelic counterparts, *in vivo* immunogenicity has not yet been confirmed; although for 36 alternative allelic variants of MiHAs, predicted HLA, HLA class I binding affinity is similar to the affinity of the respective MiHA (11). For a comprehensive review of currently known MiHAs and mechanisms of their immunogenicity see Griffioen et al. (12).

With a few exceptions, each MiHA is presented by only one HLA allele. Thus, for the immune response to occur, the donor and the recipient should not only differ in the allelic variants of MiHA-coding nsSNP but also have the restricting HLA allele. HLA-A^*^02:01 is the most frequent MHC class I allele in Caucasians, with up to 50% of individuals bearing this allele (13). Therefore, a substantial part of the patients undergoing HSCT in Europe and North America are HLA-A^*^02:01 positive. Approximately a third of the MiHAs discovered so far are restricted by the HLA-A^*^02:01. We selected 20 HLA-A^*^02:01-restricted MiHAs for our study (Table 1).

**Table 1:** HLA-A^*^02:01 restricted MiHAs, selected for genotyping panel.

**#**	**MiHA**	**Gene**	**Ch**.	**Nt**.	**AA**	**Pmm**	**Var. ID**	**References**
1	HER-2/NEU	*ERBB2*	17	**C**/G	**P**/A	0.247	rs1058808	(14)
2	HA-1/A2	*ARHGAP45*	19	G/**A**	R/**H**	0.246	rs1801284	(15)
3	HA-2	*MYO1G*	7	**C**/T	**V**/M	0.050	rs61739531	(16)
4	UTA2-1	*KIAA1551*	12	**T**/C	**L**/P	0.234	rs2166807	(17)
5	LB-ADIR-1F	*TOR3A*	1	**T**/C	**F**/S	0.250	rs2296377	(18)
6	LB-CLYBL-1Y	*CLYBL*	13	G**/T**	D/**Y**	0.056	rs17577293	(19)
7	C19ORF48	*C19ORF48*	19	T/**A**	T/**S**	0.082	rs3745526	(20)
8	TRIM22	*TRIM22*	11	C/**T**	R/**C**	0.019	rs187416296	(21)
9	LB-PRCP-1D	*PRCP*	11	T/**G**	E/**D**	0.226	rs2229437	(22)
10	LB-SSR1-1S	*SSR1*	6	A/**G**	L/**S**	0.246	rs10004	(22)
11	LB-WNK1-1I	*WNK1*	12	G/**T**	M/**I**	0.237	rs12828016	(22)
12	T4A1	*TRIM42*	3	**C**/A	**A**/E	0.202	rs9876490	(23)
13	HA-8	*PUM3*	9	**C**/G	**R**/P	0.206	rs2173904	(9)
14	LB-HIVEP1-1S	*HIVEP1*	6	A/**G**	N/**S**	0.175	rs2228220	(24)
15	LB-NISCH-1A	*NISCH*	3	**C**/T	**A**/V	0.220	rs887515	(24)
16	UGT2B17/A2	*UGT2B17*	4			0.123	esv3600873,4	(25)
17	LB-CCL4-1T	*CCL4*	17	T/**A**	S/**T**	0.246	rs1719152	(26)
18	LB-NCAPD3-1Q	*NCAPD3*	11	C/**T**	R/**Q**	0.130	rs12292394	(26)
19	LB-NDC80-1P	*NDC80*	18	G/**C**	A/**P**	0.241	rs9051	(26)
20	WDR27-1L	*WDR27*	6	**A**/G	**L**/P	0.205	rs4236176	(27)

*Ch, chromosome, bearing a MiHA coding region; Nt, nucleotide substitution; AA, amino acid substitution; P_MM_, the probability of MiHA mismatch for unrelated donor-recipient pair; Var. ID, accession number for the SNP in the dbSNP database (https://www.ncbi.nlm.nih.gov/SNP/) or for copy number variation, in the Ensembl database (http://www.ensembl.org); Ref., reference. The allelic variant and the corresponding amino acid, for which the immunogenicity is confirmed is shown in bold. UGT2B17/A2 is caused by gene deletion*.

Nonsynonymous SNPs can be genotyped by a vast arsenal of SNP-genotyping methods, with each having its limitations. The allele-specific PCR (AS-PCR) (28) and the analysis of restriction fragments length polymorphism (RFLP) (29) require an electrophoresis step to make the allele call. High resolution melting PCR (HRM-PCR) is hard to multiplex, could be unsuitable to certain SNP types and is prone to inaccuracies (30). qPCR with hydrolysis probes is accurate and fast, but the probe binding depends on the SNP allele. Most of the commercially available qPCR-based SNP genotyping kits are designed to genotype one SNP per test.

Sequence-based methods are expensive and time-consuming. The Sanger sequencing is the most accurate method, yet it lacks multiplexing. SNP genotyping could be scaled up by a single nucleotide extension reaction or by next-generation sequencing (NGS). Although NGS could be used to genotype many SNPs simultaneously, it is excessive for the small panel of known MiHAs. However, the NGS has been used for novel MiHA discovery (22, 31). SNP genotyping techniques are reviewed in Kim and Misra (32). The genotyping of MiHAs is reviewed in Spierings and Goulmy (33).

Because the number of discovered MiHAs is limited, it is not practical to either genotype SNPs one by one or to use NGS. Besides, due to the HLA restriction, it is preferable to develop the genotyping panels grouped by the HLA allele. In this study, we aimed to design a straightforward, yet robust genotyping method based on a combination of the AS-PCR and the qPCR, for the largest group of MiHA-encoding polymorphisms, restricted by HLA-A^*^02:01. We designated it AS-qPCR. Our method is a significant improvement over the previously suggested panel for MiHA genotyping (28).

## Materials and Methods

### DNA Samples

Peripheral blood samples for genotyping were obtained from HSC recipients and their corresponding donors. Blood samples of healthy volunteers were used for cloning the control plasmids. All participants signed the informed consent approved by the ethical committee at the National Research Center for Hematology, Moscow, Russia. DNA was extracted using the Wizard Genomic DNA Purification Kit (Promega, USA).

### Primers and Probes Design

**Figure 1:**
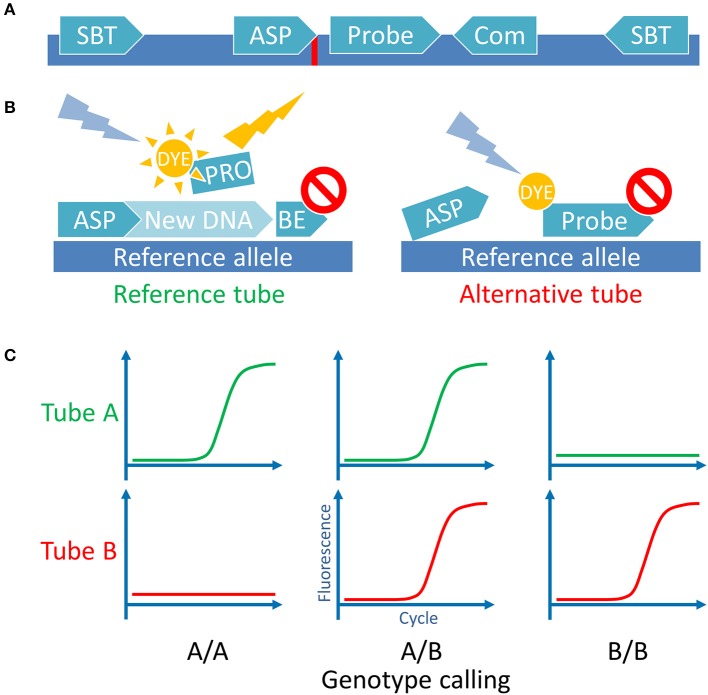
Assay principle. **(A)** Schematic representation of the MiHA-coding locus with all the utilized oligonucleotides. SBT, sequence-based typing primers used for Sanger sequencing and for control plasmids cloning; ASP, allele-specific primers used for the AS-qPCR genotyping, complementary with the 3′-end nucleotide to each of the SNP alleles (indicated by red line); Probe, a hydrolysis probe, bearing fluorescent dye and quencher; Com, common primer, used for both SNP alleles AS-qPCRs (for some SNPs common primer was used as the second SBT primer). For UGT2B17/A2 the ASP primers were used as the SBT primers. **(B)** Schematic representation of the AS-qPCR reaction. The assay is performed in 2 separate tubes with different ASPs for the same SNP. Each tube contains the common gene-specific primer (not shown) and the gene-specific fluorescent probe. Here is represented the genotyping of a DNA sample homozygous for a reference allele. **(C)** Schematic representation of the possible outcomes of the AS-qPCR and their interpretation. Allele calls are listed below the graphs.

Sequences spanning 500 bp on either side of a target SNP were extracted from the Ensembl database (http://ensembl.org, Human genome assembly GRCh38) using in house made Perl script. Other polymorphisms with frequencies more than 1% according to the dbSNP database (http://ncbi.nlm.nih.gov/SNP/, build 150) were mapped to these sequences. The Geneious gene browser 4.8 (34) was used to manage sequences and for the primers and probes design. The ASP direction was picked according to the optimal GC content, Tm, low dimer and hairpin probability. The hydrolysis probes, when possible, were designed to anneal to the same strand as the ASP, between the ASP and the common primer. We tried to position the probe as close to the ASP 3′-end as possible, guanines at the 5′-end of the probe were avoided. Several ASP variants were designed for every MiHA with introduced deliberate mismatches 1 or 2 nucleotides from the 3′-end (35, 36). We tested all ASPs together with the gene-specific probes and the gene-specific primers using the control plasmids. For each MiHA we picked the ASPs with largest qPCR Cq difference between the target allele and the opposite allele. To avoid *UGT2B17* paralog amplification both selected primers were sequence-specific, they flank the exon 6 fragment of the gene which encodes UGT2B17/A02 MiHA. All other SNPs, except MiHA-coding, were avoided during the primer and probe design. In the case of HA-8, it was impossible to avoid SNP at the probe binding site, so degenerate nucleotide was introduced. Also, for HA-1 and CCL-4 obstructive SNPs (rs3764653 and rs1049807, respectively) were in close proximity to the target SNPs and they had to be included in the ASP. We checked the linkage disequilibrium for the SNPs of interest and the obstructing SNPs and found that in both cases adjacent SNPs were linked, so we designed the ASPs accordingly. All primers and probes were tested *in silico* for dimer and hairpin formation using the IDT OligoAnalyzer tool with “qPCR” parameters setting (http://idtdna.com/calc/analyzer). All primers were designed to have a Tm in the range of 63–68°C and the probes—in the range of 70–74°C according to the Multiple Primer Analyzer tool. Resulting primer pairs and probes were checked for mispriming on the human genome with the Primer-BLAST tool (http://ncbi.nlm.nih.gov/tools/primer-blast/). Several primers were adapted from other studies studies, to some we introduced mismatches. The set of primers and probe, specific for beta-2 microglobulin (*B2M*), was used as the internal qPCR control. See Supplementary Table 1 for oligonucleotide sets and their source (if applicable). Overview of the primer positioning scheme is presented in Figure 1A.

### Control Plasmids

The NCBI Primer-BLAST tool was used to design primers flanking the genotyping primers for cloning and sequence-based typing. In several cases, the common AS-qPCR primer was used for cloning. To clone the *UGT2B17* exon six fragment we used the genotyping ASPs. First, we genotyped volunteers using AS-PCR without established positive control. For each cloning, preferably, homozygous DNA samples were selected. PCR for cloning was performed using the AccuPrime Pfx DNA polymerase (Thermo Fisher Scientific, USA) according to manufacturer's instructions. The list of cloning primers provided in Supplementary Table 2. The PCR products were purified using agarose gel electrophoresis and the GeneJET Gel Extraction Kit (Thermo Fisher Scientific, USA) and ligated into the pJet1.2 blunt vector with the CloneJET PCR Cloning Kit (Thermo Fisher Scientific, USA). The plasmid DNA from the transformed DH5α cells was extracted with the GeneJET Plasmid Miniprep kit (Thermo Fisher Scientific, USA). For the rare TRIM22 MiHA coding SNP allele we used mutagenic primers and cloning primers to perform two-step overlap extension PCR, its product was cloned as described (see Supplementary Table 3 for primer sets). All control plasmids were Sanger sequenced using standard pJet1.2 primers. The plasmids were mixed in the groups of 4 in a panel-wise manner (see below) with the concentrations equalized at 0.5–1 pg/μl to be used as the control for the genotyping panels.

## AS-qPCR

The primers and probe set combinations were tested for heterodimer formation using the Multiple Primer Analyzer tool (Thermo Fisher Scientific, https://www.lifetechnologies.com) with the default settings. Genotyping sets were pooled into 5 panels of 4 least cross-reactive primer sets. The control set for *B2M* was included in each pool. All panels were divided into 2 groups: with the ASPs for the reference and for the alternative SNP alleles, according to the reference human genome (http://ensembl.org, Human genome assembly GRCh38). The UGT2B17/A02 MiHA primers were the same for both oligonucleotide mixes. Table 2 shows the color channels assignment and the pooling scheme. For the CFX96 real-time PCR detection system (Bio-Rad, USA), used for the study, we have chosen FAM, HEX, ROX, Cy5, and Cy5,5 hydrolysis probe dyes for color channels 1–5, respectively. The BHQ-1 quencher was used for the FAM and HEX labeled probes and the BHQ-2 for the remaining dyes. Primers and probes were synthesized by EvroGen or Syntol (Russia).

**Table 2:** MiHA genotyping mixes. Primer sets are grouped into 5 multiplex panels.

**Color channel**	**Panels**				
	**I**	**II**	**III**	**IV**	**V**
I	HER-2/NEU	LB-ADIR-1F	LB-PRCP-1D	HA-8	LB-CCL4-1T
II	HA-1/A2	LB-CLYBL-1Y	LB-SSR1-1S	LB-HIVEP1-1S	LB-NCAPD3-1Q
III	HA-2	C19ORF48	LB-WNK1-1I	LB-NISCH-1A	LB-NDC80-1P
IV	UTA2-1	TRIM22	T4A1	UGT2B17/A2	WDR27-1L
V	*B2M*	*B2M*	*B2M*	*B2M*	*B2M*

*Color channel, qPCR machine filter sets. FAM, HEX, ROX, Cy5, and Cy5.5 dyes were used for first to fifth channels*.

300 nM of each primer and 200 nM of each probe (150 and 100 nM for the control set, respectively) were used for the reaction. Ready-made 5x qPCRmix-HS PCR mix (EvroGen, Russia) containing Taq polymerase was used to make the stock reaction solutions. The qPCRmix-HS qPCR mix provides 3 mM Mg^2+^ and 0.2 mM of each dNTP in the final 10 μl reaction. The AS-qPCRs were performed using two-step qPCR protocol: for 2 min, then 40 cycles of alternated 98°C for 10 s and 61°C for 30 s with plate read. The method scheme is shown in Figure 1B.

### Analysis

The SNP calling was performed using the CFX Manager 3.1 software (Bio-Rad, USA). Quantification cycle (Cq) was used to evaluate the AS-qPCR results, the Cq < 30 was considered as positive (37). We also evaluated the *B2M* fluorescence curve shape to check for possible evaporation. Related wells for the same sample and for the same MiHAs were evaluated simultaneously, target by target, starting with the internal control evaluation—respective *B2M* Cqs should be < 1.5 cycles apart. The SNP allele calling was performed according to the scheme in Figure 1C. If both PCRs were positive—the sample was marked as heterozygous, if only one well had positive signal or the signal plot crossed the threshold two or more cycles ahead of the other well—the sample was designated homozygous. Samples with the fluorescence level below 300 relative fluorescence units (RFU) were not taken into account. Both wells for the *UGT2B17* locus contain the same oligo set for that gene, so only the bi-allelic deletion will render both reactions negative.

**Figure 2:**
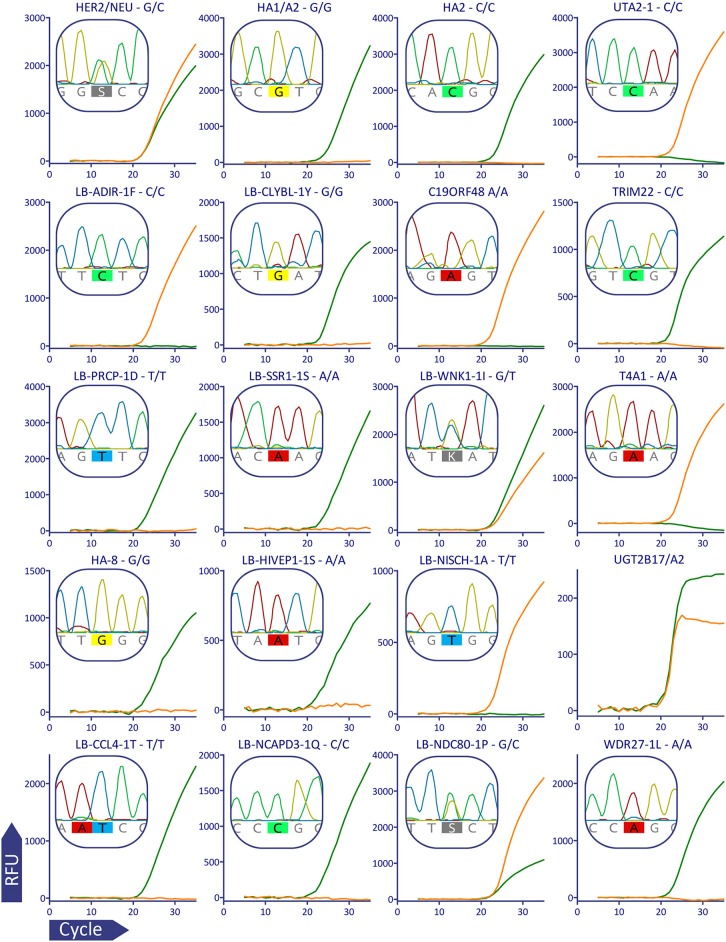
Validation of MiHA AS-qPCR genotyping for a representative subject (p909). For better visualization data from the multiplex was separated: each row corresponds to the MiHA group and each column corresponds to the color channel. Each plot shows the combined data for the two AS-qPCRs performed in the separate wells, the AS-qPCR-based SNP allele call is denoted above each plot, Sanger sequence-based allele call is shown in the insert. The fluorescent curves are in green and orange for the reference and alternative alleles, respectively. It was confirmed by Sanger sequencing that *UGT2B17* gene, and not its paralogs, is amplified in the AS-qPCR. The curves for the AS-qPCR internal control are omitted.

For plots in Figure 2 raw qPCR data were extracted in the CSV format, fluorescent curves were plotted using GraphPad Prism version 5.03 (GraphPad Software, USA, www.graphpad.com), it was also used for Supplementary Figures 1–3.

The probability of MiHA Mismatch (P_MM_) for related and unrelated donor-recipient pairs was calculated using the SNP allele frequencies for European population from the reference genome database (http://ensembl.org, Human Genome Assembly GRCh38) according to the formulas provided in Bykova et al. (38), Armistead et al. (23). The distribution of the number of the mismatches was calculated by 10^7^ iterations of simulation with an in-house python script.

### Sequencing

The PCRs were performed with the 5x qPCRmix-HS PCR mix (EvroGen, Russia) or the Phusion Hot Start II DNA Polymerase (Thermo Fisher Scientific, USA) using the cloning primers. The product was checked with agarose gel electrophoresis and PCR was enzymatically cleaned using the Illustra ExoStar kit (GE Healthcare, USA) or with electrophoresis and the gel-extraction Cleanup kit (EvroGen, Russia). The sequencing reactions were performed with the BigDye 1.1 or 3.1 chemistry, purified with the BigDye XTerminator (Applied Biosystems, USA) and sequenced using the Nanophor-05 (Syntol, Russia) or the ABI PRISM 3100 (Applied Biosystems, USA) machines. Base calling was performed using the Sequencing analysis software version 5.3 (Applied Biosystems, USA). Obtained reads were aligned with the respective reference sequences, described above, using the Geneious software version 4.8. Chromatogram images were made using Geneious version R11 (34).

The exome libraries were made using the Ion AmpliSeq Exome RDY Kit 1 × 8 (Thermo Fisher Scientific, USA) for 8 donor-recipient pairs. Exome sequencing was performed at the Research Center for Medical Genetics, Moscow, Russia using the Ion S5 system (Thermo Fisher Scientific, USA). Exome assembly was performed using the Torrent Suite software (Thermo Fisher Scientific, USA). The MiHA coding polymorphisms were analyzed using the Integrative Genomics Viewer (39). The data points with coverage below 15 reads were excluded, MiHAs C19ORF48 and LB-NDC80-1P were fully excluded due to this criterion. For the *UGT2B17* exon six coverage was analyzed.

## Results

### HLA-A^*^02:01-Restricted MiHA Panel

For our study, we have selected 20 previously described HLA-A^*^02:01 restricted MiHAs (Table 1). Most of them were discovered by the forward immunology approaches, so their ability to induce the *in vivo* immune response was confirmed. To our best knowledge, for HER2/Neu, HIVEP1-1S, NISCH-1A, UGT2B17/A2, and WDR27-1L only the *in vitro* immunogenicity was shown. UGT2B17/A2 MiHA is caused by gene deletion, so it lacks an allelic counterpart, for the other MiHAs, encoded by SNPs, both peptides are translated. For HA-1, HA-2 and HA-8 it was shown that the alternative peptides are not presented by the MHC due to impaired HLA-A^*^02:01 binding or altered antigen processing (9, 15, 29). For LB-CLYBL-1Y, LB-SSR1-1S, NDC80-1P, and LB-NISCH-1A it was demonstrated by mass-spectrometry that the allelic counterpart was presented by HLA-A^*^02:01 molecules (10, 27). If mismatched, they may elicit an immune response, but in the current study we called mismatch only in the cases where the recipient had the allele known to be immunogenic *in vivo* and the donor was homozygous for the alternative allele. Although UGT2B17/A2 was initially reported to be presented by HLA-A^*^02:06 (40), it is assumed that it may also be presented by HLA-A^*^02:01, as these alleles have similar peptide binding motifs (41). The peptide was labeled as a weak binder for both HLA-A^*^02:01 and A^*^02:06 alleles by the NetMHC 4.0 algorithm (42).

The probability of a donor and recipient to have a MiHA mismatch (P_MM_) for a particular MiHA depends on the allele frequencies (23, 38). P_MM_ in the European population ranges from 2% for TRIM22 to 25% for LB-ADIR-1F (Table 1). Using P_MM_ for each MiHA, we have calculated the distribution of the number of mismatches in related and unrelated pairs for the 20 MiHAs considered in this paper (Supplementary Figure 1). Related allo-HSCT-pairs were most likely to have 2 mismatches (28.8%), while the number of mismatches in unrelated pairs peaks at 3 (22.9%). This confirms the clinical relevance of the genotyping for the selected panel, as most transplantations would be mismatched for one or more of the studied MiHAs.

### AS-qPCR Assay Design

To achieve PCR multiplexing and to reduce the analysis time we used the combination of allele-specific PCR and real-time PCR: the allele-specific primers were used to discriminate SNP alleles while the fluorescently labeled hydrolysis probes distinguished the loci (Figure 1). With the proposed design, the genotyping of four SNPs required two separate wells: one for the detection of the reference and other for the alternative alleles. Each had the fifth, common oligonucleotide set, serving as the internal control. This design allowed for the 4-fold reduction of the number of reactions compared to a singleplex AS-PCR and the 2-fold reduction compared to a singleplex qPCR with allele-specific probes.

The ΔCq between the specific and the non-specific reaction for ASPs of our design was at least 4.9 (Supplementary Figure 2). Using the plasmid mixes, we checked the sensitivity of the allele discrimination to the amount of input DNA across the series of dilutions from 100 pg to 10 fg of plasmid DNA (pDNA) per test, which approximated to 80 μg and 0.8 ng of human genomic DNA (gDNA), respectively. We found that although in the multiplex reaction with the 5x qPCRmix-HS qPCR mix some MiHAs can be genotyped using as low as 10 fg of pDNA, reliable results were obtained with at least 1 pg of pDNA (Supplementary Figure 3). This corresponds to 80 ng of human gDNA.

### Method Validation

To validate the genotyping panel, we selected 5 HLA-matched HSCT donor-recipient pairs bearing the HLA-A^*^02:01 allele (1 sibling donor and 4 unrelated donors). All samples were genotyped in a blind manner with the reported method and using Sanger sequencing. In all of the 200 tested data points, Sanger sequencing confirmed the allele calls made with the AS-qPCR. Figure 2 shows qPCR curves and Sanger sequencing data for a representative subject, the rest of the genotyping data could be found in the Supplementary Dataset.

To further test our approach, we performed the full exome sequencing for 8 additional HLA-matched HSCT donor-recipient pairs on the Ion S5 platform. We found that some SNPs in our panel were poorly covered by the full exome sequencing (Supplementary Figure 4). Altogether, 42 points had to be excluded from the analysis due to low coverage, including all data points for C19ORF48 and LB-NDC80-1P. The remaining 278 points were compared to AS-qPCR genotyping data. We found the discrepancies in 5 cases. The SNP encoding the LB-WNK1-1I was wrongly genotyped in subject p1032 due to the rare SNP rs56245971 located 7 nucleotides upstream from the target SNP, which interfered with the ASP binding, leading to the wrong allele call. The interfering SNP was not taken into account during the design process, due to its low frequency of 0.005% (according to The Exome Aggregation Consortium, http://exac.broadinstitute.org). The remaining 4 genotyping errors were contained in panel 4 (3 for HA-8, and 1 for LB-HIVEP1-1S). We assume that they were caused by the mistake in the preparation of the genotyping mixes resulting in low signal levels. We repeated the AS-qPCR for this panel in the same blind manner and found no discrepancies. Taking this into consideration we propose that the test results with the signal level below 300 RFU should not be taken into account.

Sibling pairs have 0–5 mismatches and unrelated pairs–0 to 8 mismatches. The donor-recipient genotypes and the imputed mismatches are listed in Table 3.

**Table 3:** MiHA genotyping for 13 HLA-matched HSCT pairs.

**Pair Type**		**HER-2/ NEU**	**HA-1/ A2**	**HA-2**	**UTA2-1**	**LB-ADIR-1F**	**LB-CLYBL-1Y**	**C19ORF48**	**TRIM22**	**LB-PRCP-1D**	**LB-SSR1-1S**	**LB-WNK1-1I**	**T4A1**	**HA-8**	**LB-HIVEP1-1S**	**LB-NISCH-1A**	**UGT2B17/ A2**	**LB- CCL4-1T**	**LB- NCAPD3-1Q**	**LB- NDC80-1P**	**WDR27-1L**	**Total MM**
	Ref.	C	G	C	T	T	G	T	C	T	A	G	C	C	A	C	Im	T	C	G	A	
	Alt.	G	A	T	C	C	T	A	T	G	G	T	A	G	G	T	No	A	T	C	G	
	Imm.	C	A	C	T	T	T	A	T	G	G	T	C	C	G	C	Im	A	T	C	A	
R	p908	G/C	A	C/T	C/T	T	G	A	C	T	A	T	A	C	A	C/T	Im	T	C	G/C	A	5
	p909	G/C	G	C	C	C	G	A	C	T	A	G/T	A	G	A	T	Im	T	C	G/C	A	
U	p207	G	A	C	T	C	G	T	C	G/T	A	G/T	A/C	C	A	T	Im	T	C	G	A/G	3
	p208	G	A/G	T	C	T	G	T	C	G/T	A/G	G/T	A	G/C	A/G	T	Im	T	C/T	G	A/G	
U	p180	C	A/G	C	C	C/T	G/T	A	C	T	G	G	A	C	A/G	T	No	A/T	C/T	G	A	8
	p181	G/C	G	T	T	C/T	G	T	C	T	A	G/T	A/C	G/C	A	C/T	Im	T	C	G	A	
U	p298	G/C	G	C	C	C/T	G	A/T	C	T	A/G	G/T	A/C	G	A	C/T	Im	T	C	G	A	3
	p299	G/C	A/G	C/T	C/T	C	G	A/T	C	T	A	G	A/C	G/C	A	C/T	Im	T	C	G	A/G	
U	p444	G	A/G	C	C/T	C	G	A/T	C	T	A/G	G/T	A	G/C	A	C	Im	T	C/T	G	A/G	4
	p198	C	A	C/T	C/T	C/T	G	T	C	T	A/G	T	C	G	A	T	Im	T	C	G	A/G	
U	p1031	C	A/G	C/T	C	C	G	T	C	T	A/G	G/T	A/C	G	A	C/T	Im	T	C	G	A/G	5
	p1032	C/G	G	T	C	T/C	G	T	C	T	A	G/T	A	G/C	A	C/T	N/O	A/T	C	G	A	
R	p1138	G	A/G	C	C	C	G	A/T	C	G/T	A/G	G/T	A	G	A	C	Im	T	C	G	A	0
	p1139	C/G	A/G	C	C	C	G	A	C	G/T	A/G	G/T	A/C	G	A	C	Im	A/T	C	G	A	
R	p1056	G	G	C	T/C	T	G	A/T	C	T	A	G	A	G/C	A	T	NO	A	C	G	G	2
	p1057	C/G	G	C	T/C	C	G	T	C	G/T	A	G/T	C	G/C	A	T	NO	A/T	C	G	G	
R	p1136	G	A/G	C	T/C	C	G	A/T	C	T	A	G/T	C	G/C	A	T	Im	T	C/T	G	A/G	3
	p1137	G	A/G	C	T/C	T/C	G	T	C	T	A/G	G/T	C	G	A	T	Im	T	C	G/C	A	
U	p1151	C	A/G	C	T/C	C	G	A/T	C	T	A	G	A/C	G	A/G	C/T	Im	A/T	C	G/C	A	8
	p1152	G	A/G	T	C	C	G	A/T	C	T	A/G	G	A	G/C	A	T	Im	T	C/T	G	A/G	
U	p1161	G	A/G	C/T	T/C	T/C	G	A/T	C	T	A/G	G/T	A/C	G/C	A	T	Im	T	C	G	A	3
	p1162	C/G	A/G	C	T/C	T/C	G	A	C	T	A/G	G/T	A	G	A	T	Im	A/T	C/T	G	G	
U	p1155	C/G	A/G	T	C	C	G	T	C	T	A	G	A	G/C	A	T	NO	T	C	G	A/G	0
	p1156	C/G	A/G	C	T/C	T	G	A/T	C	T	A	T	A	G/C	A	C	Im	T	C	G	A/G	
U	p1175	G	A/G	C/T	C	C	G	T	C	T	A	T	A/C	G/C	A	T	Im	T	C	G/C	A	2
	p1176	C/G	A/G	C	C	T/C	G	T	C	T	A	T	A	C	A	T	Im	T	C	G	A	

*Ref, reference; Alt, alternative nsSNP allele; according to the human reference genome (http://ensembl.org, Human Genome Assembly GRCh38), Imm, the allelic variant with confirmed immunogenicity. Recipients and corresponding donors are listed in pairs, with patients above the donors. R, sibling; U, unrelated donor. The immunogenic mismatches underlined. Total MM, the number of immunogenic mismatches for a given HSCT pair*.

## Discussion

Here we report the method for genotyping 20 MiHA-encoding polymorphisms based on AS-PCR combined with qPCR. This approach is faster than the conventional AS-PCR, lacks the electrophoresis step and could be multiplexed. We demonstrated that up to 4 AS-qPCRs, plus the internal control, can be performed in a single tube without the loss of accuracy, and the test is robust on the wide range of gDNA concentrations.

Current work describes the genotyping of all currently known HLA-A^*^02:01-restricted MiHAs. There could be other MiHAs presented by HLA-A^*^02:01 yet to be discovered. Besides, other alleles, including HLA-A^*^01:01, A^*^03:01, A^*^24:02, B^*^07:02, B^*^08:01, and B^*^44:02, which are common in the European population, have associated MiHAs. We believe that this approach could be further extended to all immunogenic polymorphisms. Designing the genotyping kits based on the MiHA-restricting HLA allele, in our opinion, is more practical than whole exome sequencing or large SNP-genotyping panels.

The limitation of the reported approach is shared with other methods that use ASPs, i.e., previously unknown or rare polymorphisms falling into primer binding sites may affect the results. Indeed, using Sanger sequencing, we discovered a novel SNP in the ASP binding site for SNP rs9876490 (T4A1) in the sibling pair p908/p909. However, as both donor and patient were homozygous for the genotyped SNP, the novel polymorphism did not preclude the correct allele call. In subject p1032 analyzed by NGS, we found a rare SNP in the ASP binding site for LB-WNK1-1I. This SNP led to the wrong allele call by our method. These risks should be taken into consideration, but due to the high number of rare variants, it seems impractical to consider them during ASP design.

We aimed to identify mismatches that could induce the immune response in the genotyped donor-recipient pairs. For HA-1, HA-2, and HA-8 (9, 15, 29) it was demonstrated that only one of the allelic variants yielded an MHC-associated peptide, other MiHAs in this work may be immunogenic in both directions (co-dominant). In a recent study using quantitative mass-spectrometry, it was demonstrated that the allelic counterparts of LB-CLYBL-1Y, LB-NISCH-1A, and LB-SSR1-1S were presented by the MHC at comparable levels to the MiHA-encoding alleles (10). The *in vivo* immunogenicity of the alternative allelic variants still needs to be confirmed. In our assay the allelic variants are grouped according to the reference human genome and not by immunogenicity. In this way, the proposed method would remain applicable irrespective of the notion of MiHA-immunogenicity.

MiHA can contribute to the outcome of HSCT. Autosomal MiHA mismatches increased the incidence of relapse-free survival after HLA-matched sibling transplantations (43). It was recently reported that mismatches for two HLA-A^*^02:01-restricted MiHAs: HA-1 and HA-8 increased the incidence of severe acute GvHD when the donor had A/A genotype in rs231775 of *CTLA4* gene (44). The contribution of mismatches of the other MiHAs to the clinical outcome has not yet been demonstrated. The availability of the assay allowing for rapid MiHA genotyping of donor-recipient pairs should facilitate the study of the allogeneic immune response directed against MiHAs. Besides, the proposed approach can be easily adapted for genotyping other DNA polymorphisms, including SNPs in immunoregulatory genes.

Another possible application of MiHA genotyping is a selection of therapeutic targets for post-transplant immunotherapy. Up to 58% of patients relapse post-HSCT (45). MiHAs represent the attractive targets for posttransplant cell therapy as, unlike tumor neoantigens, they are germline-encoded and relatively common in the population, so all cancer cells, expressing MiHA-encoding gene, can be targeted. To avoid the potential off-tumor toxicity, immune therapy should be restricted only to MiHAs encoded by the genes selectively or predominantly expressed in the hematopoietic tissue (5). The AS-qPCR could be used for the preliminary patient and donor screening for the targetable MiHA mismatches.

We hope that the reported method will foster research of the allogeneic immune response and development of the novel immunotherapies.

## Ethics Statement

The study was conducted in accord with the Declaration of Helsinki (1964). All subjects participated in the study signed a written informed consent approved by the ethical committee at the National Research Center for Hematology, Moscow, Russia.

## Author Contributions

DR designed the method. DR, AP, and AK performed method validation and genotyping. DM performed statistical calculations and data analysis. DR and GE wrote the manuscript. All authors read and reviewed the manuscript.

### Conflict of Interest Statement

Patent application No.2017122175 describing the current method was filed with the Russian Patent Office. DR, AP, and GE had filed a Russian patent application, RU 2 675 597 C1 describing the current method. The remaining authors declare that the research was conducted in the absence of any commercial or financial relationships that could be construed as a potential conflict of interest.
