# Supplementary figures and images for: Rapid Multiplex Genotyping of 20 HLA-A*02:01 Restricted Minor Histocompatibility Antigens
Source: Front Immunol. 2019 Jun 4;10:1226. doi: 10.3389/fimmu.2019.01226 (PMC6593292; doi:10.3389/fimmu.2019.01226)

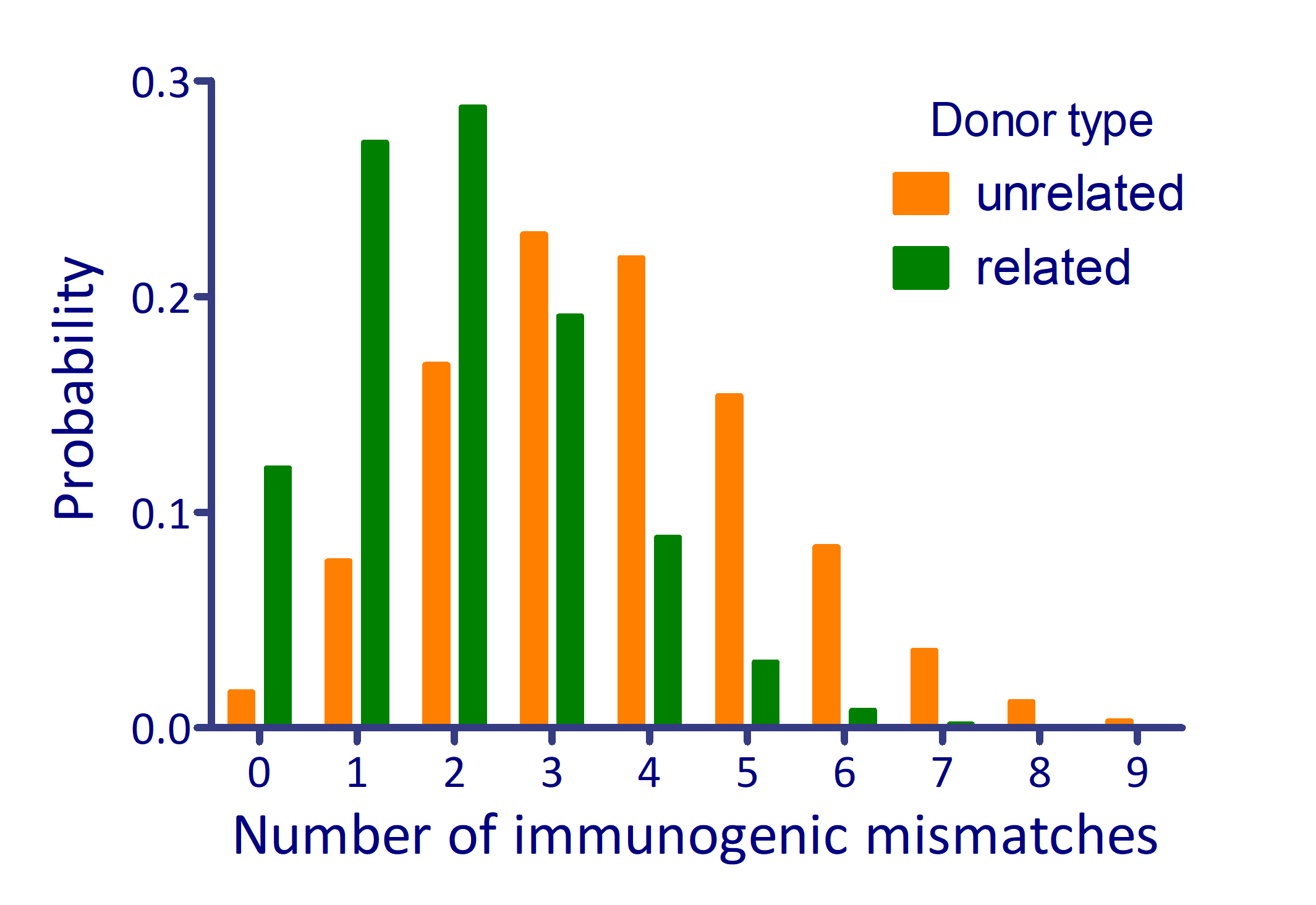

Supplement: Supplementary file 1 [file Image_1.TIF]

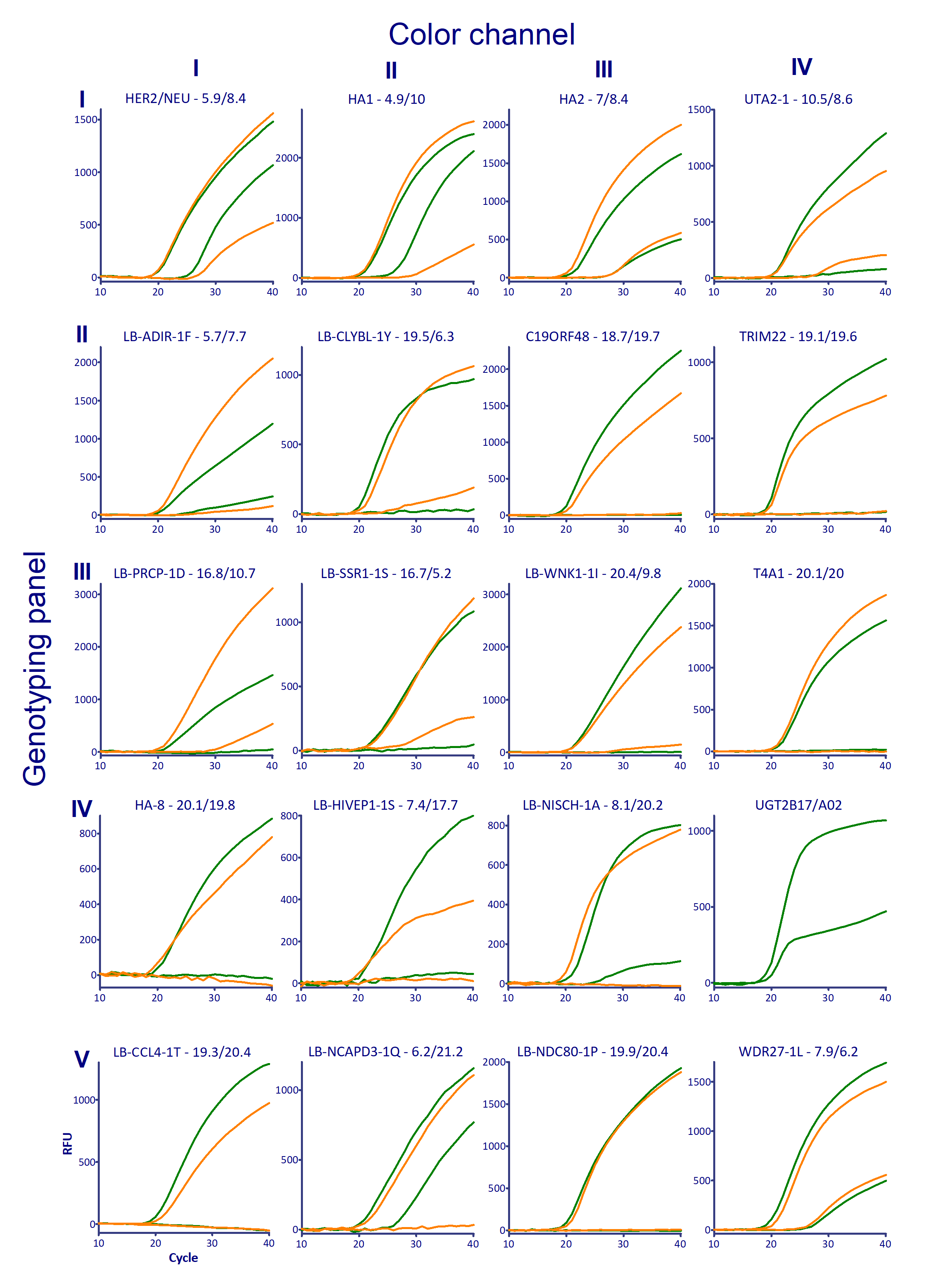

Supplement: Supplementary file 2 [file Image_2.TIF]

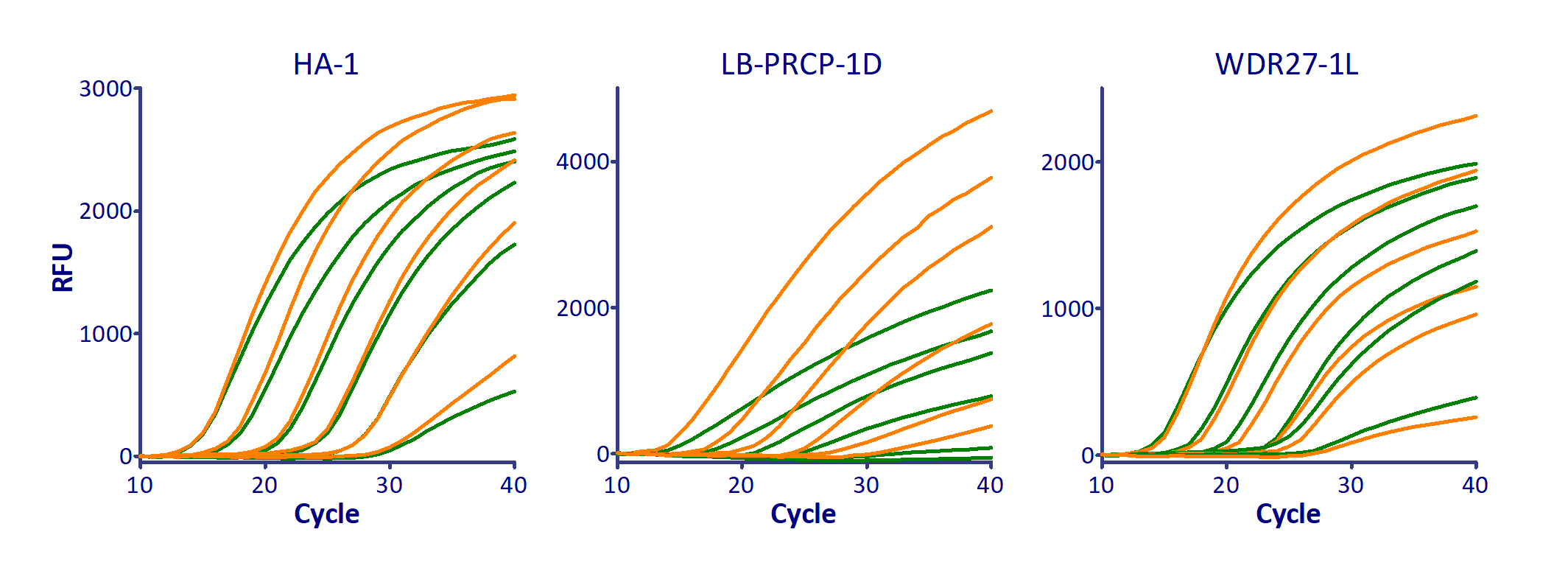

Supplement: Supplementary file 3 [file Image_3.TIF]

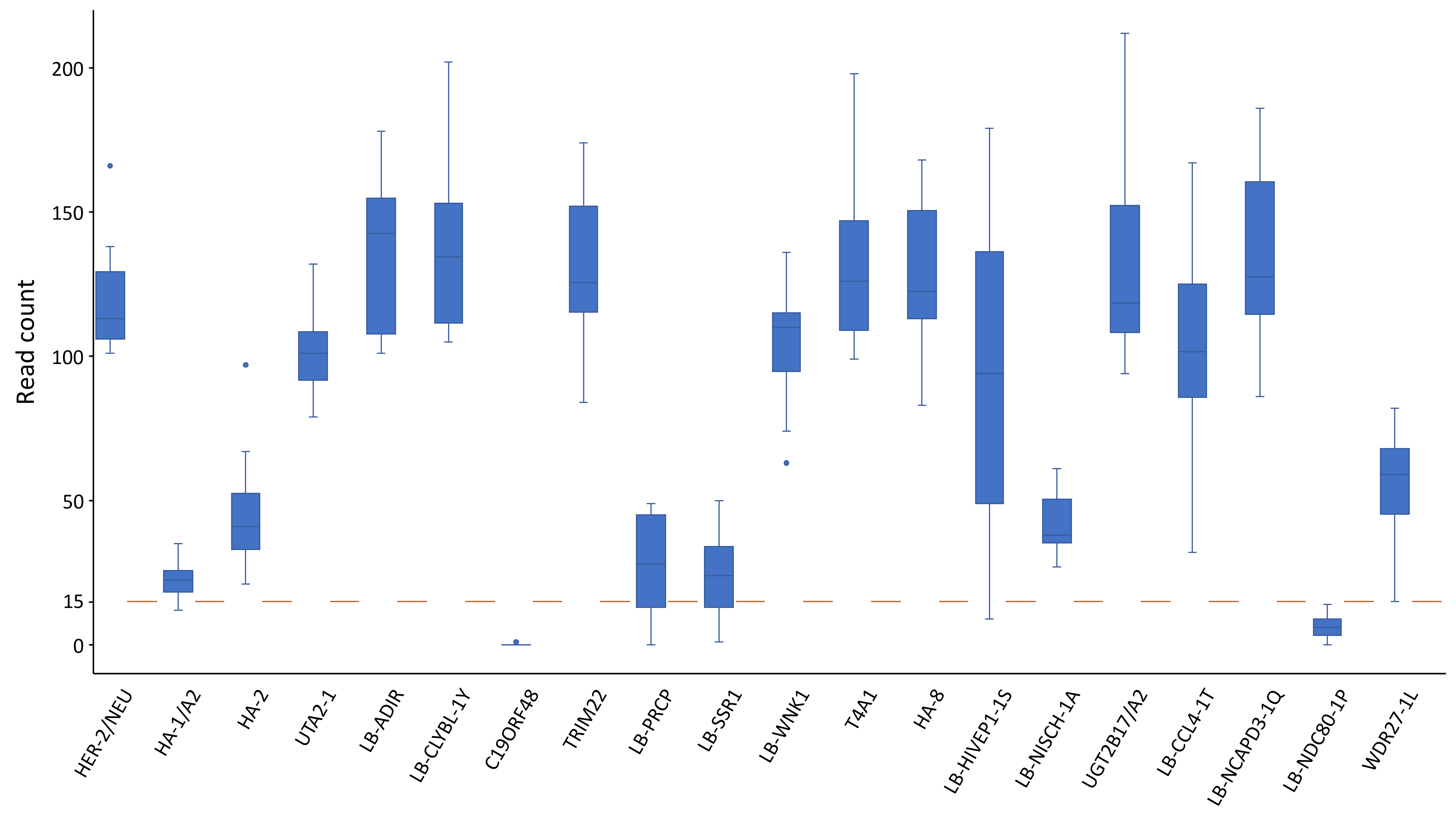

Supplement: Supplementary file 4 [file Image_4.TIF]
