# Supplementary material for: Rapid Multiplex Genotyping of 20 HLA-A*02:01 Restricted Minor Histocompatibility Antigens
Source: Front Immunol. 2019 Jun 4;10:1226. doi: 10.3389/fimmu.2019.01226 (PMC6593292; doi:10.3389/fimmu.2019.01226)
Supplement: Supplementary file 8 [file Presentation_1.pdf]

Supplementary Material

Rapid Multiplex Genotyping of 20 HLA-A*02:01 Restricted Minor Histocompatibility Antigens

Dmitrii Romaniuk[1], Anna Postovskaya[1], Alexandra Khmelevskaya[1], Dmitry Malko[1], Grigory A. Efimov[1*]

[1]Laboratory for Transplantation Immunology, National Research Center for Hematology, Moscow, Russia

***Correspondence:**
M.D., Ph.D. Grigory Efimov
efimov.g@blood.ru

1 Supplementary Data

Supplementary data is included as the archive. It includes Sanger sequencing files covering all the MiHA coding loci for 10 DNA samples used for validation, and AS-qPCR data files in the proprietary Bio-Rad format and the universal qPCR format – RDML.

2 Supplementary Figures and Tables

2.1 Supplementary Figures

Supplementary Figure 1. The number of *in silico* predicted immunogenic mismatches calculated using the MiHA-coding SNP frequencies for the European population from the reference genome database (http://ensembl.org, Human Genome Assembly GRCh38) according to the formulas provided in (1).

Supplementary Figure 2. Verification of the genotyping mixes. Control plasmids were grouped by genotyping panels and mixed 1 pg of each plasmid per reaction. Each plot shows combined data from 4 wells: specific and non-specific AS-qPCR on reference (green) and alternative (orange) alleles. Only one replica of duplicate reaction is shown. ΔCq between the specific and the non-specific AS-qPCRs, averaged for two replicas, was at least 4.9 cycles and is indicated above each plot, (reference/alternative). Rows represent the genotyping panels, columns represent the color channels. For UGT2b17/A2 gene deletion all AS-qPCRs contained the same ASP and control plasmid, so only specific AS-qPCRs are shown. RFU – relative fluorescence units.

Supplementary Figure 3. Control plasmid titration (100 pg to 1 fg) for 3 representative MiHAs. Cq decreased as the plasmid concentration increased. Only one replicate is shown. Reference alleles are shown in green, alternative – in orange. RFU – relative fluorescence units.

Supplementary Figure 4. The NGS read coverage for the 20 genomic variations studied. The box plot shows the variance of reads number covering the genomic coordinate of each SNP. For the

UGT2B17/A02 number of reads for exon 6 is plotted (samples with the bi-allelic *UGT2B17* deletion were excluded). Red dashed line indicates the threshold of 15 reads per SNP.

2.2 Supplementary Tables

Supplementary Table 1. Primers and hydrolysis probes for HLA-A*02:01 restricted MiHA genotyping. ASP – allele-specific primers. Deliberate mismatches are shown in lowercase. Each genotyping panel is divided into two sets: ASPs to the reference and to the alternative SNP alleles, according to the reference human genome (http://ensembl.org, Human Genome Assembly GRCh38).

Supplementary Table 2. Primers used for cloning. Italics indicate that AS-qPCR primers were used for cloning. For all SNPs two control plasmids were done using the same primer set, one plasmid was done for UGT2B17/A02 MiHA.

Supplementary Table 3. Primers used for TRIM22 immune (alternative) allele cloning. We used the overlap extension PCR (substitution in mutagenesis primers is shown in lowercase) in combination with the cloning primers (italicized) and gDNA sample, homozygous for the reference allele.

3 References:

1. Bykova NA, Malko DB, Efimov GA. In silico analysis of the minor histocompatibility antigen landscape based on the 1000 Genomes Project. *Frontiers in Immunology* (2018) **9**:1819. doi:10.3389/fimmu.2018.01819
